# Improved respiratory motion tracking through a novel fiducial marker placement guidance system during electromagnetic navigational bronchoscopy (ENB)

**DOI:** 10.1186/s13014-019-1306-0

**Published:** 2019-07-11

**Authors:** Hayley Stowe, Stella Ogake, Sunil Sharma, Suzanne Kelly, Morgan McDonald, Kayla Stanley, Paul Walker, Hyder Arastu, Carlos Anciano Granadillo, Mark Bowling, Andrew Ju

**Affiliations:** 1 Department of Radiation Oncology, The Brody School of Medicine at East Carolina University, 600 Moye Blvd, Greenville, NC 27834 USA; 2 Department of Internal Medicine, Brody School of Medicine at East Carolina University, Greenville, NC USA; 3 Department of Cardiovascular Sciences, Brody School of Medicine at East Carolina University, Greenville, NC USA

**Keywords:** Fiducial, Electromagnetic navigational bronchoscopy, SABR, Stereotactic ablative radiotherapy, Lung cancer, CyberKnife, SBRT

## Abstract

**Background:**

Stereotactic ablative radiotherapy (SABR) is a treatment option for patients with early stage non-small cell lung cancer (NSCLC) and recurrent or oligometastatic disease who are not surgical candidates. Due to the continuous motion of tumors within the lungs, implementing a strategy to track the target lesion is crucial. One method is to place fiducial markers which the robotic SABR system is able to track during treatment. However, placing these markers in a manner that maximizes tracking efficacy can be challenging. Using a novel fiducial placement guidance system (FPGS) during fiducial deployment may offer a way to improve the quantity of fiducials tracked by the robotic SABR system.

**Method:**

This was an institutional, retrospective review identifying all patients who received robotic SABR for lung tumors from May 2015 until January 2017. The FPGS was instituted in May 2016. The median number of fiducials tracked and the rate of complication was compared between patients whose fiducials were placed using FPGS versus those that were not.

**Results:**

A total of 128 patients with 147 treated lung lesions were identified. Of the lesions that utilized FPGS (*n* = 44), 28 had 2 tracked fiducials (63.6%), 14 had 3 (31.8%) and 2 had 4 (4.6%). Of the lesions treated without FPGS (*n* = 103), 5 had 1 tracked fiducial (4.9%), 91 had 2 (88.4%), 6 had 3 (5.8%), and 2 had 4 (1.9%). A significant improvement in the median number of fiducials tracked per fraction was observed for the lesions with fiducials placed using FPGS on Wilcoxon rank sum test (*p* < 0.001). The rate of complication was low and not statistically different between cohorts (*p* = 0.44).

**Conclusions:**

The FPGS can be used during the deployment of fiducial markers and may increase the number of fiducials tracked.

**Trial registration:**

An exemption for this retrospective review was granted by the East Carolina University IRB under UMCIRB 15-001726.

## Background

The preferred treatment for patients with early stage NSCLC is surgical resection. However, comorbidities such as chronic obstructive pulmonary disease and cardiovascular disease exclude an estimated 25% of these patients from being candidates for lobectomy [1]. In the past, those patients who were deemed inoperable were typically offered conventional radiotherapy or observation. Those who chose conventional radiotherapy had a 60–70% chance of failure to control the primary tumor [2–4]. Patients who chose observation had a more than 50% chance of dying from cancer progression [5, 6]. Unfortunately, the 2-year survival for either approach is less than 40%. With the development of SABR, it became possible to deliver highly conformal, high-dose radiation to target lesions. SABR was found to be a major improvement on conventionally fractionated radiotherapy for non-operable, early stage NSCLC patients, and offers a much higher primary tumor control rate of as high as 97.6% at 3 years [6]. Due to the elliptical motion of the lungs during the respiratory cycle, tumor motion has been a major technical obstacle [7].

There are differing strategies that are used to account for tumor motion during SABR in lung tumors: to immobilize the target (via breath holding techniques or abdominal compression), to gate the beam so that it is only delivering in a certain portion of the patient’s breathing cycle, or to actively track the target during breathing motion [8]. The robotic SABR system utilizes active tracking by adjusting for changes in target position throughout the respiratory cycle [9]. This delivery technique tracks the target either via tumor visualization using orthogonal radiographs that match intensity pattern variation of the target during treatment or by relying on fiducial markers [10]. Compared to the other strategies of accounting for target motion, our center felt more confident with a tighter expansion from tumor to planning target volume with the active tracking of a robotic system. However, in order for robotic SABR to be safe and effective, good fidelity in tracking has to be ensured. At our institution, pulmonologists utilize electromagnetic navigational bronchoscopy (ENB) to place fiducial markers. In order to best track the tumor in a variety of conditions, fiducials must be positioned in, or in close proximity to, the target area so that their movement coincides with the target’s motion [11]. As long as there are at least three fiducials tracked, the robotic SABR can potentially track lesions in 6D and adjust the treatment frame of reference. Our institutional preference is to place one fiducial within the tumor, or at the biopsy site when placement is combined with a biopsy, and the others around the tumor. Ideally, fiducials should be > 2 cm apart from each other and form an angle of > 15 degrees for 6D tracking [12, 13].

Once the fiducial markers are in place, an imaging system that utilizes the two orthogonal x-ray sources in conjunction with amorphous silicon detectors acquires live imaging of the patient during treatment, allowing for real-time imaging of the fiducials. Superficially placed infrared light-emitting diodes (LEDs) on the abdomen are monitored on a separate camera array. The tracking system then creates a predictive model where the motion of the fiducials on the orthogonal x-rays through the phases of the breathing cycle is correlated with the breathing phase data derived from the infrared LEDs. This enables the robotic SABR to continuously predict the motion of the fiducials and tumor via the movement of the LEDs and to ensure that the treatment beam is on target despite system latency [13]. This system requires a minimum of 1 fiducial to track the translational motion of the tumor, and at least 3 fiducials for 6D tracking of the translations and rotations of the target [12].

Inserting fiducials with minimal complications may be difficult. The bronchoscopic technique has been proven to have a more desirable side effect profile when compared to the other two options [14–16]. For example, when using the CT-guided percutaneous placement method via an 18-gauge needle, Bhagat et al. found a pneumothorax rate of 67% [17]. When using a 19-gauge needle for the same approach, Kothary et al. had a 45% pneumothorax development rate [18]. The ENB modality is an all-inclusive and minimally morbid management strategy for inoperable, early-stage NSCLC patient population [19]. In a study performed by Harley et al. analyzing the efficacy of endobronchial ultrasound and ENB-based fiducial placement, only one of the 48 patients included in the study developed a pneumothorax [20]. Furthermore, unlike other techniques, ENB affords the opportunity to stage the mediastinum via endobronchial ultrasound, to biopsy the primary tumor, and to place fiducials all during a single procedure. Though achieving all three goals during one procedure lengthens the time to completion, it is thought that this three-in-one method could expedite management of lung cancer and reduce the overall rate of complications [21].

ENB has improved upon the standard bronchoscopic method for the placement of fiducials in thoracic tumors. ENB is an image-guided approach that uses a 3D-reconstructed CT-scan and an electromagnetic field board to access peripheral lung lesions beyond the reach of conventional bronchoscopes in real time [22]. The American College of Chest Physicians (ACCP) guidelines have recommended ENB for the evaluation of peripheral lung lesions that cannot be reached by conventional bronchoscopy [23]. This system combines the strengths of three tools in order to reach its target. Firstly, a CT scan is obtained in order to recreate a 3-D virtual reconstruction of the airways. Secondly, an eight-way steerable probe that possesses a position sensor is piloted through the endobronchial tree. Lastly, an electromagnetic board is connected to a computer containing the planning data. This board is able to track the location of the probe tip and relay the information to the treatment console being viewed by the pulmonologist, thus giving the operator real-time positional information within the bronchial tree [24].

**Fig. 1:**
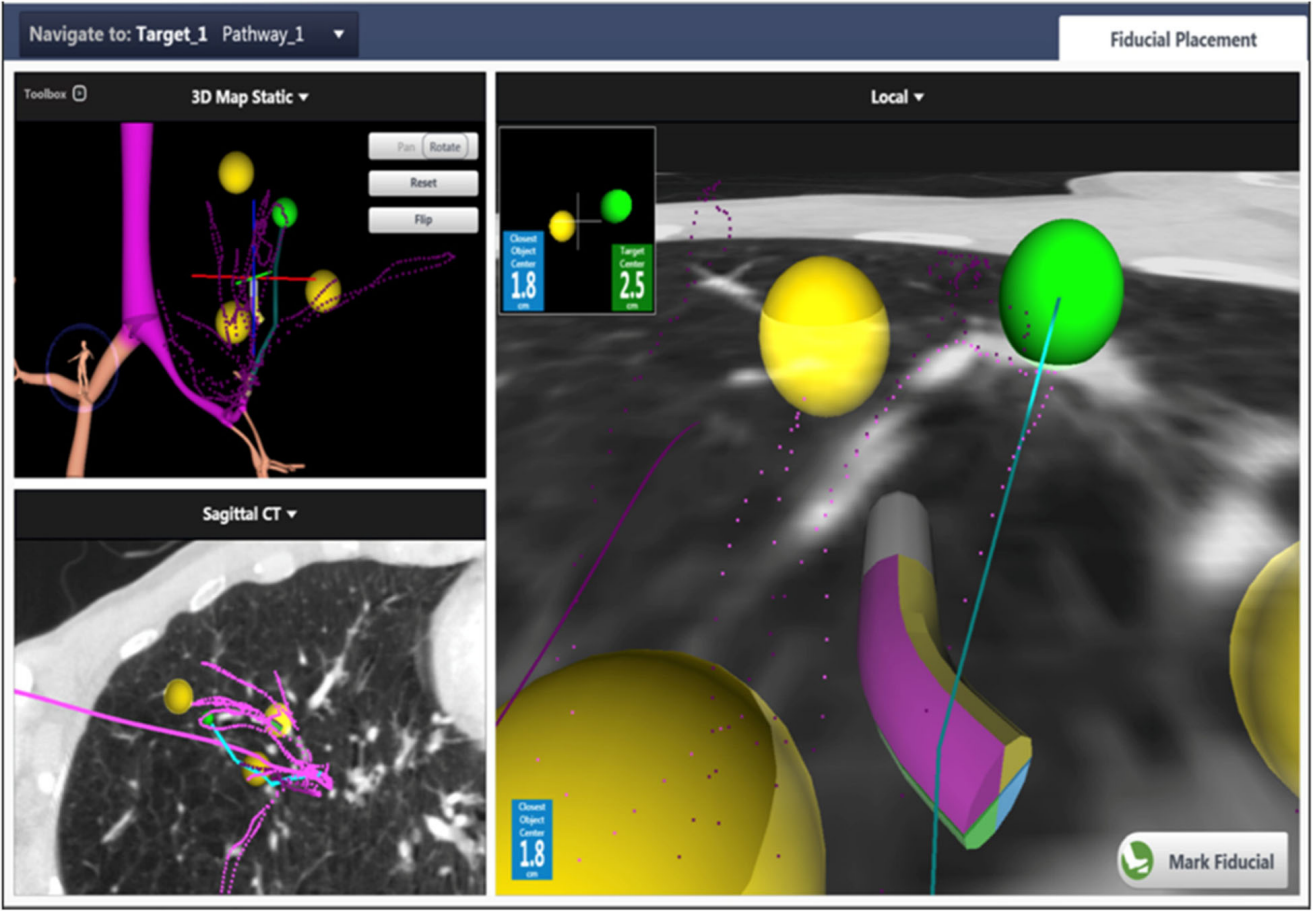
Fiducial placement guidance system example. A screenshot from the FPGS system during the fiducial placement procedure. The green sphere represents the tumor and suggested targets for placement of a fiducial as specified by the FPGS are marked in yellow

A novel fiducial placement guidance system (FPGS) was developed for use during an ENB procedure. The FPGS suggests target locations for fiducial placement that are in an optimal geometry for detection on orthogonal x-rays acquired by the robotic SABR delivery system (Fig. 1). These locations are generated after surveying the region around the tumor for fiducial placement targets that are accessible by bronchoscope. The FPGS creates a “map” for the pulmonologists to navigate to these suggested locations and to place fiducials at their ideal parameters of > 2 cm apart from each other and form an angle of > 15 degrees for 6D tracking [11]. The fiducial locations are calculated to be in smaller airways to minimize fiducial migration.

This study will focus on analyzing the results obtained with and without the use of FPGS to determine whether the implementation of the placement system had a positive impact on fiducial tracking in the robotic SABR system in our institution. The primary study aim was to assess the efficacy of the FPGS’ ability to increase the number of fiducials tracked during robotic SABR therapy.

## Methods

### Patients

This is an institutional retrospective review that includes all patients who received robotic SABR for lung tumors from May 2015 until January 2017. The type of lesions included in this study consist of presumptive stage I NSCLC, pathologically confirmed stage IA, IB, and IIA NSCLC, metastatic lesions to the lung from extrathoracic primary tumors, locally recurrent NSCLC, and patients previously treated with SABR who subsequently developed either a second lung primary or developed metastatic disease. Those who were considered presumptive stage I had insufficient material for definitive diagnosis or had a PET-positive lesion that could not be biopsied. Metastatic lesions were included in the FPGS cohort since the use of SABR to treat oligometastatic lung lesions became a more widely accepted practice during this time frame. We excluded patients with tumors primary located in the chest wall, patients treated with more than 10 fractions, and patients who were treated with spine tracking or fiducial-based tracking without respiratory motion management.

This study compares lesions treated by SABR where fiducials were placed using ENB with the FPGS versus lesions that did not utilize the guidance system for fiducial placement. Within the non-FPGS cohort, 2 patients had their fiducials placed under CT guidance by interventional radiology, 1 other patient had fiducials placed under conventional bronchoscopy with fluoroscopic guidance, and 1 patient had their fiducials placed at an outside institution where the method of placement could not be determined. The FPGS was instituted in May 2016. The superDimension Navigation System (superDimension, Inc., Plymouth, MN) was used in ENB, the FPGS was developed by Medtronic (Dublin, Ireland) as an add-on to the superDimension navigational system, with developmental input from one of the authors of this study, M.B. The number of fiducials tracked per fraction by the Cyberknife (Accuray, Inc., Sunnyvale, CA) Synchrony respiratory motion management system during robotic SABR was compared between lesions with fiducials placed using FPGS versus those that did not; complication rates were also compared.

**Fig. 2:**
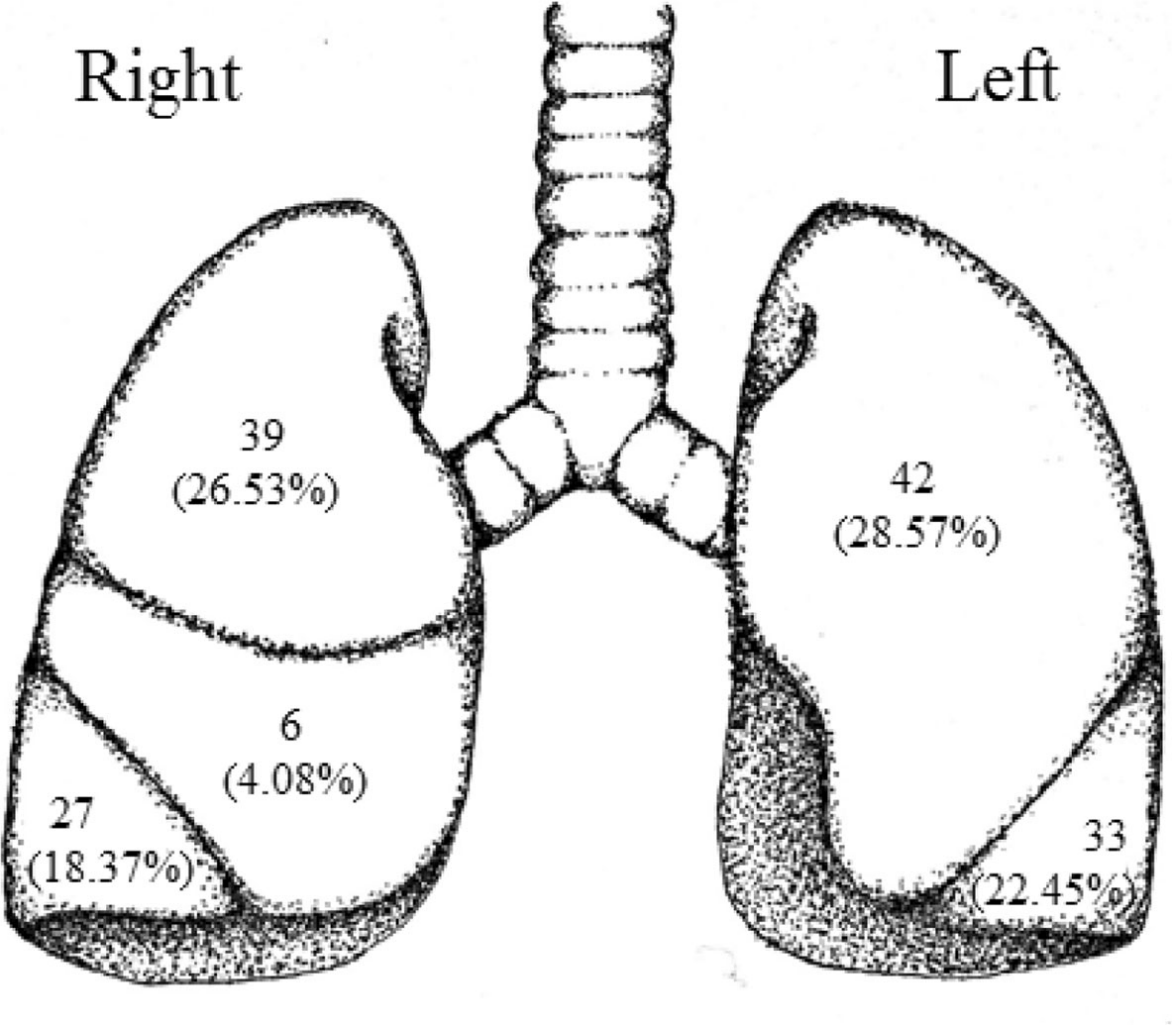
Anatomical location of lung lesions. Number of lesions, and the percent of the total number of lesions, located in each lobe of the lung

A total of 128 patients with 147 treated lung lesions were identified. Of the total 147 lung lesions, 39 were presumptive Stage I NSCLC, 29 were Stage IA, 11 were Stage IB, 3 were Stage IIA NSCLC, 41 were metastatic tumors of various primary sites, 8 were locally recurrent NSCLC, 11 were either a second primary or Stage IV NSCLC, and 5 other lesions did not fit the above categories (Table 1). The anatomical locations of the lesions included 39 right upper lobe (26.5%), 6 right middle lobe (4.1%), 27 right lower lobe (18.4%), 42 left upper lobe (28.6%), and 33 left lower lobe (22.4%) (Fig. 2). Of the total 147 lesions, 44 (29.9%) had fiducials placed by FPGS and 103 (70.1%) did not. The median fractions for both cohorts combined was 5 (range 3–10) with a median dose of 60 Gy (range 24–60 Gy). Fiducial placement related complications were defined based on the Common Terminology Criteria for Adverse Events (CTCAE) scale version 4 [24, 25].

**Table 1:** Lesion characteristics. This table categorizes the lesions based on stage and whether or not the FPGS was used

Diagnosis	Lesions with fiducials placed without FPGS	Percent non-FPGS	Lesions with fiducials placed with FPGS	Percent FPGS	Total number of lesions
Presumptive Stage I	22	21.36%	17	38.64%	39
Actual Stage IA	22	21.36%	7	15.91%	29
Actual Stage IB	8	7.77%	3	6.82%	11
Actual Stage IIA	3	2.91%	0	0.00%	3
Metastatic	30	29.13%	11	25.00%	41
Locally Recurrent	7	6.80%	1	2.27%	8
Second Primary vs. Stage IV	8	7.77%	3	6.82%	11
Other	3	2.91%	2	4.55%	5
Total	103		44		147

### Statistics

The primary study aim was to assess the ability of the FPGS to increase the number of fiducials tracked during robotic SABR therapy. Patients were divided into cohorts based on whether or not the FPGS was used. For each treated lesion, the median of the number of fiducials tracked per fraction was calculated. One patient in the non-FPGS cohort had a median of 1.5 fiducials tracked in his course of treatment, for analysis this was rounded up to 2. Clinical factors that were evaluated included cancer Stage, anatomic location of tumors, complications, number of fiducials tracked, and total number of fractions received. Descriptive analysis was used to calculate the percentages, means and medians between cohorts. The Wilcoxon rank sum test was used to test for significance, with *p* < 0.05 being considered statistically significant. Statistical calculations were conducted using the MedCalc12.6.00 (Medcalc, Mariakerke, Belgium) statistical package.

## Results

Complications for fiducials placed in lesions that used the FPGS (*n* = 44) included pneumothorax (2.27%) and bleeding (2.27%), CTCAE Grade I and II, respectively. For the lesions that did not utilize the FPGS (*n* = 103), complications included one incidence of Grade II pneumothorax (0.97%). The non-FPGS cohort did also have two incidences of hypoxia and one of hypotension. However, these events were not used in the assessment of intervention-related complications as per Trotti et al. in the development of CTCAE [24]. The rate of complication was not statistically different between cohorts (*p* = 0.44). None of the 4 patients in the non-FPGS cohort who were not recorded to have utilized ENB had a documented toxicity.

**Fig. 3:**
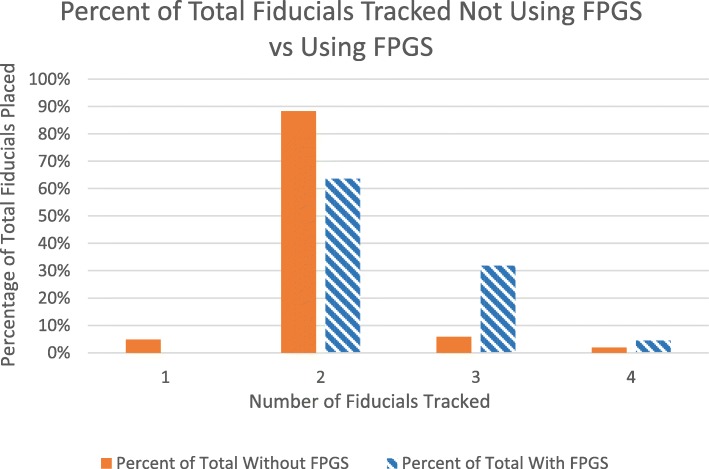
Fiducials tracked . Comparison of the distribution of the percentage of fiducials tracked in cases that did not use the FPGS (solid column) vs. cases that did utilize the FPGS (striped column)

**Fig. 4:**
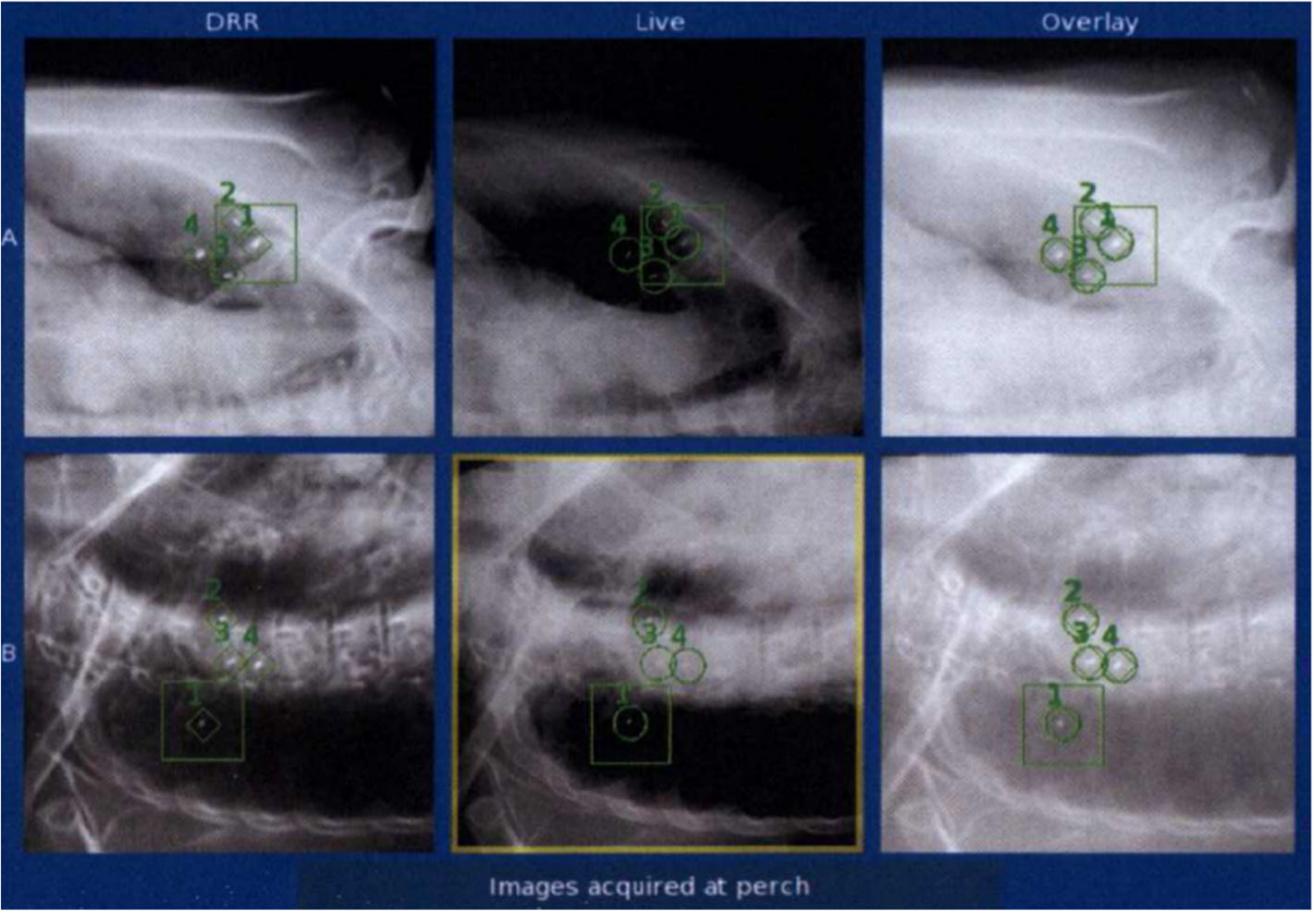
Fiducial tracking during treatment. An example of the robotic SABR system tracking 4 fiducials on orthogonal cameras during treatment

The median number of fiducials implanted using the endobronchial navigation was 4 in both cohorts (range 2–5 for FPGS, range 3–6 without FPGS, though not recorded in 12 of the patients in the FPGS cohort). Only 1 patient in the study, who was in the FPGS cohort, had less than 3 fiducials implanted. Of the lesions that utilized FPGS (*n* = 44), 28 had a median of 2 fiducials tracked by the SABR system (63.6%), 14 had 3 (31.8%) and 2 had 4 (4.6%). Of the lesions treated without FPGS (*n* = 103), 5 had 1 tracked fiducial (4.9%), 91 had 2 (88.4%), 6 had 3 (5.8%), and 2 had 4 (1.9%) (Fig. 3). An example of a patient with 4 fiducials tracked on the robotic SABR system after using the FPGS for placement can be seen in Fig. 4. A significant improvement in the median of fiducials tracked per fraction was observed in the lesions with fiducials placed using FPGS (*p* < 0.001), including an increase in the percentage of patients where 3 fiducials were tracked, which was the minimum required for potential rotational correction as well as translational correction in the robotic SABR system.

## Discussion

In our retrospective study, a total of 4.55% (*n* = 2) of the lesions with fiducials placed using ENB with FPGS developed complications, and in the non-FPGS cohort, 0.97% (*n* = 1) developed CTCAE defined complications. When analyzing all of the lesions where the fiducials were confirmed to have been placed by ENB (*n* = 143), a total of 2.04% (*n* = 3) patients in this study developed complications. We will note that the two that developed a pneumothorax were undergoing concurrent biopsy and fiducial placement. The overall rate of complications seen in this study is comparable to other studies which demonstrate that patients who receive ENB-placed fiducials are less likely to develop adverse effects when compared to the percutaneous method [16, 19].

Our study showed a significant improvement on the number of patients with at least three fiducials tracked when using the FPGS (36.36% vs 7.77%), thus increasing the possibility of 6D tracking. However, despite the improvements in the number of fiducials tracked, not all patients had three fiducials tracked, so there are still improvements to be made on the system. For example, the FPGS at its current version does not take into account the possibility that fiducials could shadow each other on the orthogonal x-rays used in the robotic SABR system when suggesting placement positions for fiducials. In addition, tracking three fiducials does not always result in 6D tracking. This is perhaps due to the more expansile nature of the lung tissue causing issues in assessing rotations, as compared to other organs that are treated with robotic SABR, such as the prostate.

**Fig. 5:**
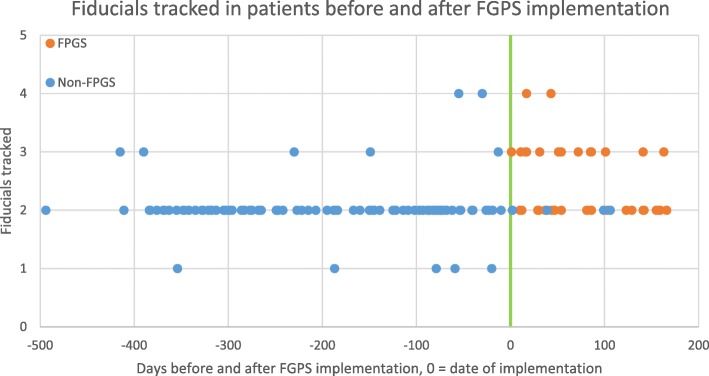
Fiducials tracked over time. Number of fiducials tracked over time, with *t* = 0 as the date of FPGS implementation, with an improvement in tracking immediately after FPGS implementation

This study is limited by its retrospective design. The retrospective nature lends to selection bias and incomplete patient, tumor, and treatment details that may otherwise be captured in a prospective study. There was also a specific date in which our institution began implementing the FPGS for most patients, which could introduce additional bias as the pulmonologists performing the procedure could gain more experience over time. However, the principle pulmonologist who placed the fiducials both before and after the initiation of the FPGS had several years of experience using ENB prior to the time period included in this cohort, and there was a steep increase in the median of fiducials tracked immediately after FPGS implementation (Fig. 5). This suggests it was not merely an accumulation of improved experience using the new system that improved tracking. The data for the number of fiducials that were implanted is missing for 12 patients in the non-FPGS system cohort, however 2 of those non-FPGS patients with missing data had 3 fiducials tracked on SABR, and the rest had 2 fiducials tracked, resulting in a mean and median for tracked fiducials in those 12 patients of 2.17 and 2 respectively. This appears to be reflective of the distribution of the remainder of the non-FPGS cohort, and we do not feel as though this would significantly affect the overall analysis of this study. Another potential confounding factor of this study is that the indications for lung SABR had been expanding over the period in which the FPGS system was implemented, particularly a relative increase over time of the patients being treated for oligometastatic disease, however the overall percentage of the different indications for SABR remained relatively comparable between the FPGS and non-FPGS cohorts, as shown in Fig. 1. Despite these limitations, this study provides important information with regards to the safety and efficacy of a FPGS when placing fiducials for patients with NSCLC.

## Conclusions

Our retrospective study showed that using the FPGS resulted in a statistically significant improvement in the median of fiducials tracked during robotic SABR therapy. Fiducial placement using the FPGS did not result in an increase in placement-related complications. Additional research is needed to continue to improve the number of tracked fiducials.

## Data Availability

The datasets used and/or analyzed during the current study are available from the corresponding author on reasonable request.

## Abbreviations

ACCP: American College of Chest Physician
CTCAE: Common Terminology Criteria for Adverse Events
CTCAE: Criteria for Adverse Events
ENB: Electromagnetic navigational bronchoscopy
FPGS: Fiducial placement guidance system
NSCLC: Non-small cell lung cancer
SABR: Stereotactic ablative radiotherapy
